# Introduction of Nanoscale Si_3_N_4_ to Improve the Dielectric Thermal Stability of a Si_3_N_4_/P(VDF-HFP) Composite Film

**DOI:** 10.3390/polym15214264

**Published:** 2023-10-30

**Authors:** Jing Guan, Laifei Cheng, Ye Fang

**Affiliations:** Science and Technology on Thermostructural Composite Materials Laboratory, Northwestern Polytechnical University, Xi’an 710072, China; betty_guanjing@163.com (J.G.); yefang511@nwpu.edu.cn (Y.F.)

**Keywords:** silicon nitride, PVDF, dielectric materials

## Abstract

In order to improve the dielectric thermal stability of polyvinylidene fluoride (PVDF)-based film, nano silicon nitride (Si_3_N_4_) was introduced, and hence the energy storage performance was improved. The introduction of nano Si_3_N_4_ fillers will induce a phase transition of P(VDF-HFP) from polar β to nonpolar α, which leads to the improved energy storage property. As such, the discharging energy density of Si_3_N_4_/P(VDF-HFP) composite films increased with the amount of doped Si_3_N_4_. After incorporating 10wt% Si_3_N_4_ in Si_3_N_4_/P(VDF-HFP) films, the discharging density increased to 1.2 J/cm^3^ under a relatively low electric field of 100 MV/m. Compared with a pure P(VDF-HFP) film, both the discharging energy density and thermal dielectric relaxor temperature of Si_3_N_4_/P(VDF-HFP) increased. The working temperature increased from 80 °C to 120 °C, which is significant for ensuring its adaptability in high-temperature energy storage areas. Thus, this result indicates that Si_3_N_4_ is a key filler that can improve the thermal stability of PVDF-based energy storage polymer films and may provide a reference for high-temperature capacitor materials.

## 1. Introduction

The advanced electronic and electrical industry requires energy storge capacitors possessing the characteristics of miniaturization, easy portability, good stability, and so on [1,2]. Thus, as a key component of capacitors, dielectric materials must be lightweight, flexible, and have a high energy storage density [3]. After decades of efforts, the traditional inorganic dielectric materials, such as barium strontium (BaTiO_3_)-series ceramics, calcium copper titanate (CCTO), and lead zirconate titanate (PZT), have been well investigated and widely used as capacitors in some areas [4]. These dielectric ceramics show the characteristics of a large Young’s modulus, a high dielectric constant (ε_r_), and low dielectric loss (tan δ), but they have a low energy density and weak tolerance for changing electric fields, and additionally, these ceramics are hard, brittle, and difficult to process into specially shaped units [5]. More significantly, this ceramic material is unable to meet the requirements of small size, high flexibility, and portability in a high-power-density capacitor. In contrast, dielectric polymers are light, easy to process, and show considerable electrical resistance (>500 MV/m), which is believed to make up for the disadvantages of ceramics [6].

The energy density, U_e_, of dielectric materials is an important parameter to evaluate when assessing energy storage capacity. To improve the energy density of polymers, Qiming Zhang et al., in 2006 [7], proposed that the oriented polyvinylidene fluoride trifluorochloroethylene (P(VDF-CTFE) can be employed for energy storage, as it has a large electric dipole moment (-CF_2_-CH_2_-) and produces a huge electrical displacement (D) (about 0.13 μC/cm^2^) in P(VDF-CTFE [8]. After stretching, the ε_r_ of P (VDF-CTFE) is about 12, E_b_ is as high as 700 MV/m, and ***U_e_*** reaches 21 J/cm^3^ at the maximum polarization electric field, which is far higher than that of BOPP [9]. As is already known, PVDF-based polymers are nonlinear dielectrics, and ***U_e_*** is usually calculated from formula [10]

(1) $$U_{e}=\int E\;dD$$

Thus, besides displacement, using polymer materials with an ***E_b_*** larger than 500 MV/m is essential for achieving a high capacitor ***U_e_***. However, for nonlinear PVDF-based polymers, working at such a high electrical field while maintaining good stability is difficult [11]. More importantly, PVDF-based polymers have poor thermal conductivity, and multiple charging and discharging processes at high-frequency electrical fields will generate a lot of heat, resulting in an increase in the temperature of the capacitor and further leading to the thermal aging of the polymer materials or even the destruction of related capacitors [12].

To solve this problem, many researchers have proposed introducing ceramic particles, such as BaTiO_3_, PZT, copper calcium titanate (CCTO), etc., as fillers into PVDF-based polymers to construct a polymer/ceramic composite [13,14,15]. One of the purposes of this design is to improve the electrical displacement and dielectric properties by virtue of the high ion displacement of these ceramics. Another reason for choosing this design is to reduce the working electrical field of dielectric polymers. For example, recently, an energy density of 10.54 J/cm^3^ was obtained in a Ba_0.6_Sr_0.4_TiO_3_/PVDF composite thin film under 300 MV/m, which is larger than that of pure PVDF [16]. And a K_0.5_Na_0.5_NbO_3_-SrTiO_3_/PVDF polymer composite film was prepared with a recoverable energy storage density of 1.34 J/cm^3^ under 100 MV/m [17]. Moreover, BaTiO_3_-doped PVDF composites with a well-treated interface exhibit a maximum energy storage density of 4.08 J/cm^3^ under 200 MV/m [18]. In the above research, although the ***U_e_*** under a low electrical field was improved, the heat generated from frequent charging–discharging processes still posed a big problem to the use of these materials in capacitors. This is because most ceramic fillers have poor thermal conductivity [19,20].

For this reason, researchers recently proposed doping PVDF-based polymers with ceramics with high thermal conductivity, and the fillers used were mainly SiC-series inorganic particles [21,22,23]. The introduction of fillers with high thermal conductivity, to a certain degree, solved the problem of the poor thermal conductivity of composite materials. However, SiC-series inorganic particles are also good electric conductors, which can lead to considerable energy loss in PVDF-based composites. Thus, how to improve the thermal stability while maintaining an improved ***U_e_*** and low loss should be further investigated [24].

Thus, in this work, we selected the relaxor ferroelectric copolymer of polyvinylidene fluoride hexafluoropropylene (P(VDF-HFP)) as the polymer matrix, as it proved in our former report [25] to possess a favorable energy storage performance. In addition, nano-sized Si_3_N_4_ was used as a replacement for SiC-series fillers. We expected to achieve a relatively high discharging energy density with good thermal stability for this Si_3_N_4_/P(VDF-HFP) two-phase composite film.

## 2. Materials and Methods

**Figure 1:**
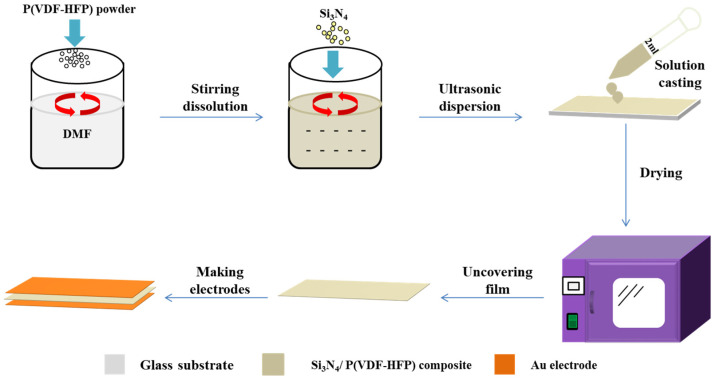
Schematic diagram of the preparation process of Si_3_N_4_/P(VDF-HFP) composite film.

Silicon nitride (Si_3_N_4_) with a size ranging from 100 nm to 200 nm was obtained from Suzhou Yuante Material Co., Ltd. (Suzhou, China), and was further purified using analytic reagent ethyl alcohol. P(VDF-HFP) powder with a 91/9 mol% VDF/HFP ratio was purchased from Piezotech Co. (Pierre-Bénite, France). Si_3_N_4_/P(VDF-HFP) composite films were prepared using the solution casting method, as shown in Figure 1. P(VDF-HFP) was dissolved in dimethyl formamide (DMF) and then purified using the filter element. After that, purified Si_3_N_4_ nanoparticles were introduced into the solutions of P(VDF-HFP) with various mass fractions of 0 wt%, 5 wt%, 7.5 wt%, 10 wt%, 12.5 wt%, 15 wt%, 17.5 wt%, 20 wt%, and 22.5 wt%. After stirring for 12 h, the uniform solution was coated on the glass slides at room temperature in the oven for the drying process. Then, the solvent was evaporated at 70 °C for 12 h, and in order to remove the DMF solvent completely, the dried Si_3_N_4_/P(VDF-HFP) composite film was melted by holding it at 170 °C for 6 h. To modify the crystalline properties of P(VDF-HFP) [26], the hot Si_3_N_4_/P(VDF-HFP) melt was cooled rapidly to room temperature in air, and similar Si_3_N_4_/P(VDF-HFP) composite films with different Si_3_N_4_ fractions and a relatively uniform thickness of 30 μm were obtained. For electrical characterization, the different films were cut into the desired dimensions and gold electrodes 80 nm in thickness were sputtered on both sides of the film as the top and bottom electrodes.

The crystal phases of Si_3_N_4_/P(VDF-HFP) composite films were examined using X-ray diffraction (XRD) with a RIGAKU D/MAX-2400 diffractometer (Akishima, Japan) with a scanning rate of 20°/min, and the wavelength of the X-ray was 1.542 Å (Cu Ka radiation, 40 kV and 100 mA). A Shimadzu Fourier 8400S spectrometer (Kyoto, Japan) was employed to scan the Fourier transform infrared (FTIR) spectra of samples in the wavelength range of 400–1500 cm^−1^ with a resolution of 0.85 cm^−1^. SEM Quanta F250 (Oberkochen, Germany) with an energy-dispersive X-ray spectroscopy (EDS) detector was used to determine the morphology of the composite films. The dielectric frequency and thermal properties were measured using an Agilent-4284A (Lake Mary, FL, USA) precision impedance analysis LCR tester in the frequency range of 100 Hz–10 MHz. A TF Analyzer 2000 ferroelectric test system (Aachen, Germany) was employed to obtain the displacement vs. electric field unipolar hysteresis (D-E) curves with a triangular voltage wave form at a frequency of 10 Hz.

## 3. Results and Discussion

**Figure 2:**
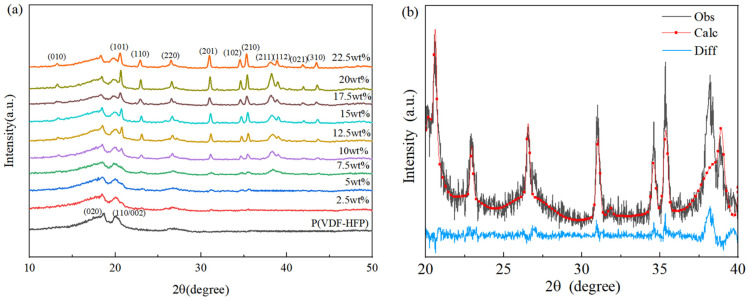
XRD pattern of Si_3_N_4_/P(VDF-HFP) composites. (**a**) XRD of different Si_3_N_4_ contents and (**b**) the refined result of Si_3_N_4_/filler.

In order to investigate the influence of the introduction of Si_3_N_4_ into the crystal structure of P(VDF-HFP), Figure 2 shows the XRD diagram of Si_3_N_4_/P(VDF-HFP) composite film samples doped with different amounts of Si_3_N_4_. The pure P(VDF-HFP) film exhibited obvious characteristic diffraction peaks at 18.5° and 20.2°, corresponding to the (020) and (110/002) crystal planes, respectively [24]. Regarding these, (020) is the typical characteristic peak of the α-crystal phase, and (110/002) are the typical characteristic peaks of the β-crystal phase. Compared with the pure P(VDF-HFP) film, the XRD of the composite film samples doped with Si_3_N_4_ exhibited not only diffraction peaks of the polymer matrix but also the peaks of Si_3_N_4_ at the positions 13.3°, 20.6°, 22.9°, 26.5°, 31.1°, 34.5°, 35.3°, 38.1°, 38.3°, 41.9°, and 43.5°, corresponding to (010), (101), (110), (220), (201), (102), (210), (211), (112), (021), (310), and other crystal planes. Moreover, Figure 2b provides the refined result of the XRD patterns of Si_3_N_4_ particles, indicating the existence of the α-crystal phase in the Si_3_N_4_ sample obtained, and the pattern matches the standard card peak of slice PDF# 41-0360 [27]. The successful recombination of P(VDF-HFP) and Si_3_N_4_ is shown, and the characteristic diffraction peak intensity of Si_3_N_4_ was seen to gradually increase with the increase in Si_3_N_4_ content. 

In addition, as the content of Si_3_N_4_ increased, the characteristic diffraction peak of the sample (110/002), corresponding to peak 20.6°, shifted to about ~20.0°. According to the Bragg formula, the increasing crystal plane spacing of the sample indicates that the addition of Si_3_N_4_ had an influence on the crystal form of P(VDF-HFP) film. Although it showed a slight change, the peak shift from plane (110/002) to plane (110) of P(VDF-HFP) corresponds to the phase transition from β to α(γ) due to the presence of Si_3_N_4_ filler. This is because the ~20.0° peak refers to the diffraction position of the α- or γ-crystal phase, according to the literature. [28] Compared with highly polar β-P(VDF-HFP), whose remnant polarization is so high that the dipoles cannot reverse with frequent electric fields, non-α-P(VDF-HFP) is more suitable as a material for high-frequency charging–discharging capacitors [29].

**Figure 3:**
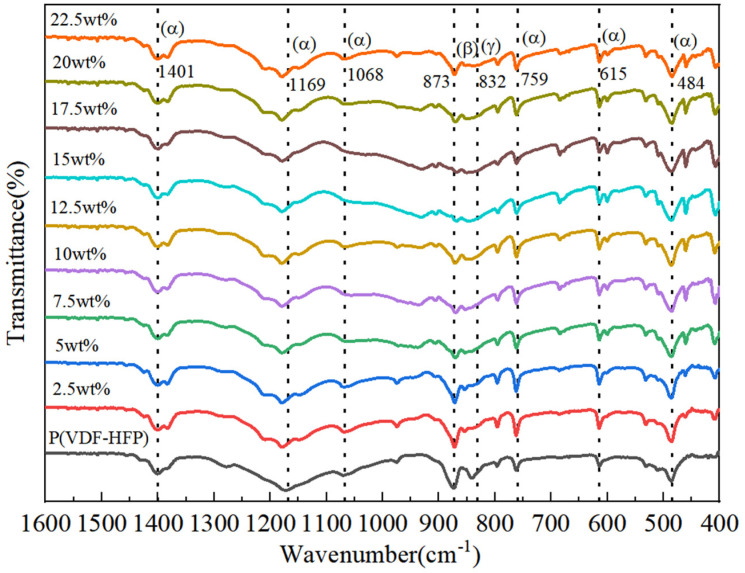
FTIR of Si_3_N_4_/P(VDF-HFP) composites with different Si_3_N_4_ contents.

To further investigate and verify the crystal structure of Si_3_N_4_/P(VDF-HFP) composite films, FTIR was used to analyze the crystal structure of composite film samples with different Si_3_N_4_ contents, as shown in Figure 3. Clearly, the absorption peaks of the α phase, β phase, and γ phase appeared in all the thin-film samples. The six absorption peaks that appeared at 484 cm^−1^, 615 cm^−1^, 759 cm^−1^, 1068 cm^−1^, 1169 cm^−1^, and 1401 cm^−1^ corresponded to the α phase (TGTG conformation of -CF_2_-CH_2_- chains); the absorption peak at 873 cm^−1^ corresponded to the β phase (TTTT); and the absorption peak at 832 cm^−1^ corresponded to the γ phase (TGTG’) [30]. Among these absorption peaks, with the addition of Si_3_N_4_, the absorption peak intensity at 873 cm^−1^ of the β phase weakened, indicating that all the trans β-crystal phases decreased with the introduction of Si_3_N_4_ particles. Moreover, with the introduction of Si_3_N_4_, the absorption peak intensity of the α phase slightly increased, and the absorption peak at 832 cm^−1^ of the γ phase disappeared. This was due to the occurrence of the phase transition from the γ phase to the α phase. Additionally, importantly, the crystallinity of P(VDF-HFP) was slightly depressed by the introduction of Si_3_N_4_, and the FTIR results are consistent with the XRD results. This phase transition promotes the dielectric and energy storage properties of this kind of two-phase composite.

**Figure 4:**
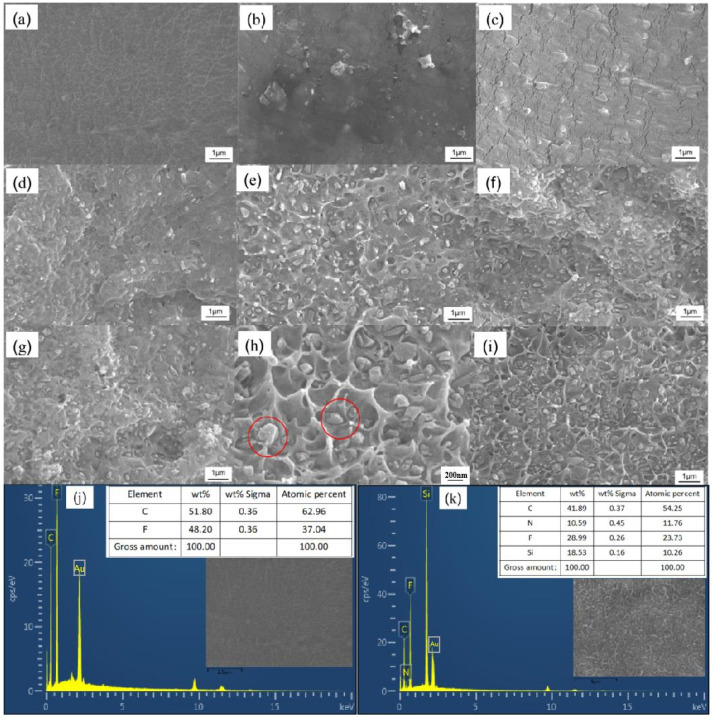
SEM images of P(VDF-HFP)-based two-phase composites with different Si_3_N_4_ contents: (**a**) 0 wt%, (**b**) 5 wt%, (**c**) 7.5 wt%, (**d**) 10 wt%, (**e**) 12.5 wt%, (**f**) 15 wt%, (**g**) 17.5 wt%, (**h**) 20 wt%, and (**i**) 22.5 wt%. EDS of (**j**) pure P(VDF-HFP) and (**k**) Si_3_N_4_/P(VDF-HFP) composite with 22.5 wt% Si_3_N_4_.

Figure 4a–i show the cross-sectional SEM images of Si_3_N_4_/P(VDF-HFP) composites with different Si_3_N_4_ doping contents, indicating that the P(VDF-HFP) matrix was successfully doped with Si_3_N_4_ nanoparticles. As the doping amount of Si_3_N_4_ increased, an increasing amount Si_3_N_4_ was observed in the polymer matrix. However, when the doping amount of Si_3_N_4_ was too high, an obvious agglomeration phenomenon appeared in some areas of the composite films, such as that seen with a doping amount of 20 wt% Si_3_N_4_, as shown in Figure 4h. In order to further verify the composite composition between the filler and the matrix, EDS detection was performed on pure P(VDF-HFP) and the composite film sample with a Si_3_N_4_ content of 22.5 wt%, as shown in Figure 3j,k. In Figure 4j, the peak positions and percentage contents of the C and F elements (H element has no inner electrons and cannot be determined) in pure P(VDF-HFP) can be clearly seen. In Figure 4k, the peak positions and percentage contents of the C and F elements in pure P(VDF-HFP) and the Si and N elements in Si_3_N_4_ can be observed, which further confirms the successful development of a composite of the filler and the matrix.

**Figure 5:**
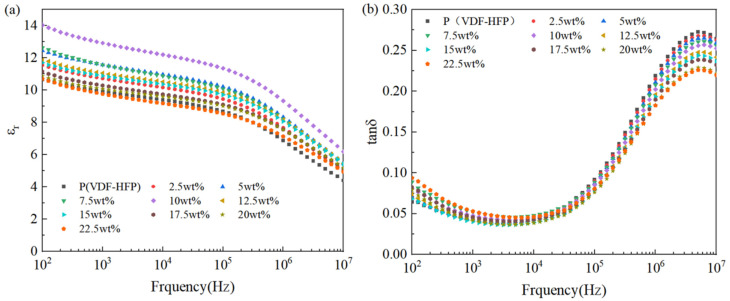
Dielectric property–frequency patterns: (**a**) ε_r_ and (**b**) tan δ.

To clarify the effect of Si_3_N_4_ on the electric properties of P(VDF-HFP)-based composite films, Figure 5a,b present the variation trend of the relative dielectric constant (εr) and dielectric loss (tanδ) of different Si_3_N_4_/P(VDF-HFP) composite film samples with frequency and Si_3_N_4_ content. Firstly, it can be seen from Figure 5a that ε_r_ decreased with increasing frequency. According to the theory of polarization, when the frequency of the AC electric field is small, various polarization modes, such as electron polarization, atomic polarization, dipole orientation polarization, and interface polarization of the dielectric material, will contribute to ε_r_. Therefore, ε_r_ is relatively high at low frequencies. As the frequency increases, the dipole orientation polarization cannot keep up with the rate of change in frequency, making it unable to play its great role, resulting in the decrease in ε_r_. As the frequency further increases and the dipole orientation polarization is further weakened, ε_r_ is greatly reduced at high frequencies. In addition, with the increase in Si_3_N_4_ content, the Si_3_N_4_/P(VDF-HFP) composite films’ ε_r_ was enhanced to a certain extent: the value of the 10wt% Si_3_N_4_ reached 14 at 100 Hz, which is 33.3% higher than that of pure P(VDF-HFP) (~10.5). The reason for the enhancement of ε_r_ is that the appropriate addition of Si_3_N_4_ increases interface polarization and ion polarization.

Moreover, variation in the tan δ of all Si_3_N_4_/P(VDF-HFP) composite film samples followed a trend of first decreasing and then increasing with the increase in frequency. In the low-frequency range, the tan δ of composite materials mainly depends on conductivity loss, which decreases with increasing frequency. In the high-frequency range, the tan δ of composite materials mainly depends on polarization loss, which increases with increasing frequency. In addition, the tan δ of Si_3_N_4_/P(VDF-HFP) composite material increases as the content of Si_3_N_4_ increases, as shown in Figure 5b, and this is because higher amounts of Si_3_N_4_ result in agglomerated defects in the P(VDF-HFP) matrix, leading to an increase in the heterogeneity of the composite material and a higher tanδ than that of pure polymers [28,31]. 

**Figure 6:**
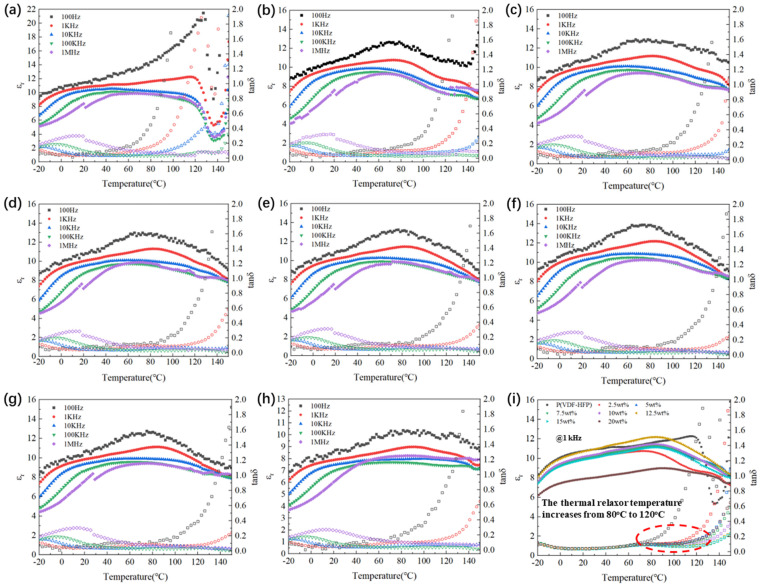
Dielectric property–temperature patterns of Si_3_N_4_/P(VDF-HFP) composites at frequencies of 100 Hz, 1 kHz, 10 kHz, 100 kHz, and 1 MHz for (**a**) 0 wt%, (**b**) 2.5 wt%, (**c**) 5 wt%, (**d**) 7.5 wt%, (**e**) 10 wt%, (**f**) 12.5 wt%, (**g**) 15 wt%, and (**h**) 20 wt%. (**i**) Dielectric property–temperature patterns of all Si_3_N_4_/P(VDF-HFP) composites at the frequency of 1 kHz.

In order to further investigate the influence of Si_3_N_4_ content on the dielectric thermal stability of Si_3_N_4_/P(VDF-HFP) composites, we determined the dielectric temperature spectrum of different samples tested at a frequency ranging from −20 °C to 150 °C, as shown in Figure 6. In pure P(VDF-HFP), the ε_r_ curve had no obvious peak (Figure 6a), and the wide dielectric peak corresponded to the dielectric relaxation generated by dipole polarization with increasing temperature. This is because the crystallization zone of pure P(VDF-HFP) is mainly a nonpolar α phase and a few polar β phases. After the appropriate addition of Si_3_N_4_, the dielectric peak became weaker as a result of the reduction in the β-phase proportion, as proved by the XRD and FTIR results. Nevertheless, the dielectric temperature spectrum curve exhibits a dielectric peak at approximately 75 °C, which was attributed to the dielectric temperature relaxation of nonlinear PVDF-based copolymers, as shown in Figure 6b–h, instead of the F-P phase transition. In addition, the ε_r_ of all film samples decreased with increasing frequency, which is consistent with the dielectric frequency spectrum.

In practical applications, the temperature stability of Si_3_N_4_/P(VDF-HFP) composite material as a functional capacitor also deserves special attention. To evaluate the dielectric temperature stability of Si_3_N_4_/P(VDF-HFP) composites, Figure 6i summarizes the dielectric temperature spectrum of all Si_3_N_4_/P(VDF-HFP) composites at a frequency of 1 kHz. As the temperature increased, the ε_r_ curves of all samples showed a trend almost parallel to the abscissa, indicating that the Si_3_N_4_/P(VDF-HFP) composite material samples had good temperature stability. The reason for this is that Si_3_N_4_/is a good conductor of heat, being able to significantly absorb the heat from the composites and improve the thermal stability and dielectric properties of Si_3_N_4_/P(VDF-HFP). As such, Si_3_N_4_/P(VDF-HFP) with a high Si_3_N_4_ content of 20wt% possessed a gentler dielectric curve than other samples, as indicated in Figure 6i. Attractively, compared with pure P(VDF-HFP), the thermal relaxor temperature of the Si_3_N_4_/P(VDF-HFP) exhibiting dielectric loss increased from 80 °C to 120 °C, which is significant for high-temperature energy storage areas.

**Figure 7:**
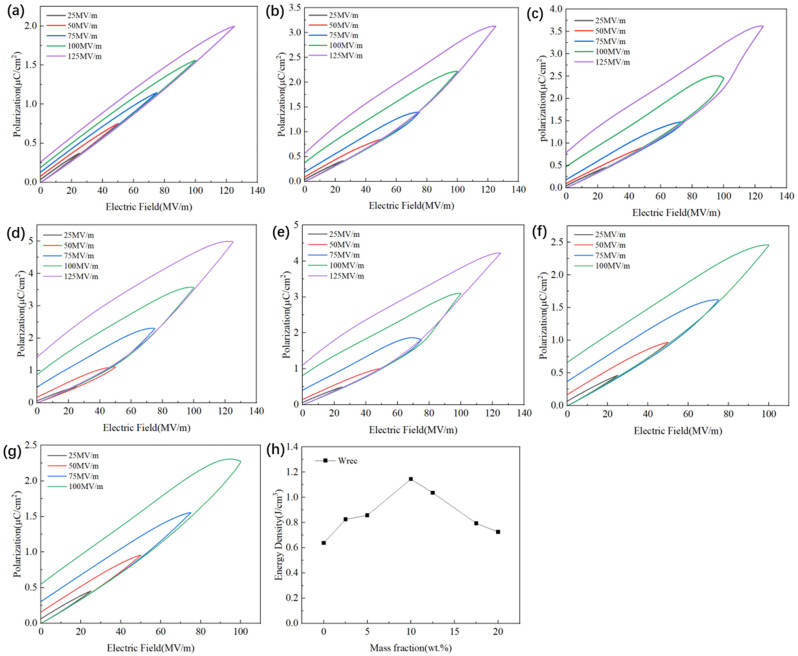
Unipolar P-E curves of Si_3_N_4_/P(VDF-HFP) composites with different Si_3_N_4_ contents: (**a**) 0 wt%, (**b**) 2.5 wt%, (**c**) 5 wt%, (**d**) 10 wt%, (**e**) 12.5 wt%, (**f**) 17.5 wt%, (**g**) 20 wt%. (**h**) W_rec_ of composite materials with different Si_3_N_4_ contents at 100 MV/m.

In addition, Figure 7a–g show the unipolar hysteresis loops of all Si_3_N_4_/P(VDF-HFP) composite film samples at a frequency of 10 Hz under an increasing electrical field. Clearly, the maximum polarization (***P_m_***) and residual polarization (***P_r_***) of all samples gradually increased with the increase in the electric field. This is because a large electric field can cause large orientation polarization in a material. As the content of Si_3_N_4_ increased, Pm and ***P_r_*** first increased and then decreased. The main reason for this is that an appropriate amount of Si_3_N_4_ can increase interface polarization and ion polarization. However, excessive Si_3_N_4_ will cause more defects in the material, leading to an unpleasant current leakage. In order to determine the energy storage performance of Si_3_N_4_/P(VDF-HFP), composite film samples were prepared by the institute as raw materials for capacitors, and the formula [8]

(2) $$W_{rec}=\int_{P_{r}}^{P_{max}}E\;dP$$

was used to calculate the recoverable energy density (***W_rec_***) of all samples. Here, E is the electric field and P is the polarization intensity. According to the formula, a large polarization difference (***P_m_-P_r_***) is the key to obtaining a large ***W_rec_***. Table 1 lists the ***P_m_***, ***P_r_***, and ***P_m_-P_r_*** of Si_3_N_4_/P(VDF-HFP) composite film samples.

Figure 7h shows the ***W_rec_*** of all Si_3_N_4_/P(VDF-HFP) composite film samples with different Si_3_N_4_ contents. As seen in Figure 7h, W_rec_ first increased and then decreased with the increase in Si_3_N_4_ content, and it reached 1.2 J/cm^3^ when the content of Si_3_N_4_ increased to 10 wt%. The reason for this increase is that the addition of an appropriate amount of Si_3_N_4_ increased ***P_m_-P_r_***, resulting in an increase in the calculated ***W_rec_***. Then, ***W_rec_*** decreased as more Si_3_N_4_ was added. The reason for this is that excess Si_3_N_4_ may lead to the current leakage and has a negative influence on the discharging energy density.

## 4. Conclusions

In this work, Si_3_N_4_/P(VDF-HFP) composite film samples were prepared using the solution casting method. The crystal structure, microstructure, dielectric property, temperature stability, and energy storage performance of the Si_3_N_4_/P(VDF-HFP) composite materials were characterized and tested. Through characterization methods such as XRD, FTIR, and SEM, it was found that a P(VDF-HFP) matrix with Si_3_N_4_ filler was successfully composed. Through analyzing the dielectric temperature spectrum, it was found that the appropriate addition of Si_3_N_4_ increased the ferroelectric phase of the P(VDF-HFP) polymer matrix, and the dielectric properties of the sample exhibited good temperature stability from −20 to 150 °C. In addition, the ***W_rec_*** of the composite film sample was obtained by calculating the unipolar hysteresis loops, and it was found that the appropriate addition of Si_3_N_4_ contributed to the improvement in energy storage performance. Therefore, the Si_3_N_4_/P(VDF-HFP) composite material proposed in this work can be applied to capacitor devices operating under high-temperature conditions.

## Figures and Tables

**Table 1:** ***P_m_***, ***P_r_*** and ***P_m_-P_r_*** of Si_3_N_4_/P(VDF-HFP) composite film samples with different Si_3_N_4_ contents.

Si_3_N_4_ Contents	*P_m_*	*P_r_*	*P_m_-P_r_*
0.0 wt%	1.56	0.19	1.37
2.5 wt%	2.23	0.38	1.85
5.0 wt%	2.5	0.47	2.03
10.0 wt%	3.57	0.87	2.7
12.5 wt%	3.1	0.81	2.29
17.5 wt%	2.45	0.67	1.78
20.0 wt%	2.3	0.55	1.75

## Data Availability

Data available on request.
